# Analysis of disordered abrasive scratches on titanium surfaces and their impact on nuclear translocation of yes-associated protein

**DOI:** 10.1038/s41598-022-26203-0

**Published:** 2022-12-15

**Authors:** Satoshi Migita, Keita Wakabayashi

**Affiliations:** Graduate School of Science and Engineering, Yamagata University, 4-3-16 Jonan, Yonezawa, Yamagata Japan

**Keywords:** Biomaterials, Biomedical engineering

## Abstract

The morphology of the metallic surface of an implant is important for its contact with bone tissue as it directly affects osteoblast functions, such as cell adhesion, proliferation, and differentiation. Firm contact between the implant and cells creates a barrier that prevents inflammation and bacterial infections. Therefore, optimizing surface morphology, such as surface roughness adjustments, is essential to improving the adhesion between the implant and cells for successful tissue regeneration. However, the manner in which the cells sense the surface roughness and morphology remains unclear. Previously, we analyzed cell adhesion behavior and observed that inhibited cell spreading can delay osteoblast functions. Therefore, assuming that the surface morphology can be sensed through cell spreading, we investigated the cell spreading area and yes-associated protein (YAP) localization in mouse osteoblasts (MC3T3-E1) on a titanium surface with disordered abrasive scratches. Surface roughness of 100–150 nm was obtained by polishing, which inhibited the cell spreading, indicating that YAP localization in the nucleus was lower than that on other surfaces. The obtained results indicate that the cells sense the surface environment based on their spreading area, which regulates cellular functions via the Hippo pathway.

## Introduction

Titanium (Ti) and its alloys exhibit excellent mechanical properties, high corrosion resistance, and non-cytotoxicity resulting in superior biocompatibility^1–4^. They are used as basic materials in various applications, including biodevices, such as implants and frameworks, tissue engineering, and regenerative medicine^1^. However, the interaction between the implant and the tissue remains inadequate. The bone–titanium contact rate is approximately 30–70%^5–7^, posing risks of implant loosening and bacterial infections^8^. To improve the implant-to-cell contact, the behavior of cells on implant devices should be analyzed to increase the success rate of implantations and treatments that use Ti-based medical devices.

Surface morphology is a critical parameter affecting cell behavior, such as adhesion, proliferation, and differentiation^8, 9^. Therefore, optimization of implant surface morphology can improve the implant-to-cell contact^10^. Acid etching and anodization techniques create micro/nano-morphology on Ti and induce higher osteogenesis at the cellular level^9^. Nano-topographical surface created by catecholic polyglycerol coating fabrication has been reported to moderate the actin fiber tension and osteogenesis on surfaces with low roughness (Ra = approximately 50 nm); however, low actin fiber tension and low osteogenesis are typically induced by surfaces with high roughness (Ra = 300–1000 nm)^11^. Crosstalk of the cell division cycle 42 protein (Cdc42), a key member of the Rho GTPase family, regulates the actin fiber formation on roughened Ti^12^. Additionally, the Wnt/β-catenin pathway is crucial for transducing the topographical cues^13^. Although the impact of surface morphology on cellular functions, such as integrin-mediated signal transduction^14–16^, has been elucidated, the method through which the cells sense surface roughness and morphology at the early cell adhesion before integrin induction or expression remains unclear.

Previously, we investigated cell adhesion behavior on Ti by creating submicron-sized morphology via polishing^17, 18^. The obtained surface inhibited the cell spreading, delaying the osteoblast functions, such as attachment, growth, and differentiation^17, 19^. Based on this analysis, we hypothesized that cells sense the surface morphology by spreading. Cell spreading is crucial in the regulation of mechanotransduction via the Hippo signaling pathway^20^. Furthermore, the yes-associated protein (YAP), a co-activator of the transcriptional enhanced associate domain, is a core component of the Hippo pathway that regulates cellular functions, such as proliferation and cell death^21, 22^.

Therefore, in this study, we investigated the cell spreading area and YAP localization in mouse osteoblasts (MC3T3-E1) on Ti with disordered abrasive scratches. The results strongly indicated that cells sense the surface morphology through their spreading and morphology, which regulates cellular functions, such as actin fiber formation via the Hippo pathway.

## Materials and methods

### Preparation and characterization of materials

Commercially sourced grade-2 pure Ti (Nilaco Corporation, Tokyo, Japan) were cut into disks of 8.0 mm diameter and 2.0 mm thickness. They were polished in a polishing machine (TDP900; South Bay Technology Inc., CA, USA) using a silicon carbide polishing paper with different grid sizes of 240, 600, and 1200; the corresponding specimens were labeled #240, #600, and #1200, respectively. The specimen prepared by mirror polishing with a colloidal silica suspension was labeled MP. After polishing, all specimens were ultrasonically rinsed in methanol and deionized water and then air-dried. The surfaces of the specimens were observed under a laser microscope (LEXT OLS4000; Olympus, Tokyo, Japan).

### Cell culture

The specimens were sterilized with 70% ethanol for 1 h under ultraviolet light and air-dried. MC3T3-E1 mouse osteoblasts, which purchased from RIKEN BRC, were seeded onto the materials in Dulbecco’s modified Eagle’s medium (FUJIFILM Wako Pure Chemical Corporation, Osaka, Japan), supplemented with 10% fetal bovine serum, 100 U/mL penicillin, and 100 μg/mL streptomycin (Nacalai Tesque, Kyoto, Japan). Osteoblasts were seeded at a density of 5,000 cells/cm^2^ on the specimens and incubated at 37 °C in a CO_2_ incubator to evaluate the cell spreading.

### Analysis of cell morphology

After 4 and 24 h of incubation, the non-adherent cells were removed by washing twice with phosphate-buffered saline (PBS). Cell morphology, actin cytoskeleton, and vinculin distribution were observed using fluorescence microscopy (Ts2-FL; Nikon Co., Ltd., Tokyo, Japan). Images were captured using a charge-coupled device (CCD) camera (WRAYCAM-NOA2000, Wraymer, Osaka, Japan) and processed using ImageJ software version 1.53e (http://imagej.nih.gov/ij/). To facilitate observation, the cells were seeded onto the materials at a density of 5000 cells/cm^2^. Actin cytoskeleton was examined by fixing the cells with 4% paraformaldehyde. After fixation, the cells were stained using Alexa Fluor 488 Phalloidin (Thermo Fisher Scientific, Waltham, MA, UCA) and Hoechst 33342 (Chemical Dojin Co., Ltd., Kumamoto, Japan) for 30 min. Subsequently, the cells were washed with PBS and observed using fluorescence microscopy. Furthermore, the cells were fixed with 4% formaldehyde for 30 min to examine the vinculin distribution. After fixation, the cells were permeabilized with 0.25% Triton X-100 in PBS for 15 min. The cells were then treated with 0.1% bovine serum albumin (BSA) to block the non-specific binding of the antibody. The cells were incubated with an anti-vinculin monoclonal antibody (1:500; Sigma-Aldrich, MO, USA) for 30 min. Subsequently, the cells were washed with PBS and incubated with Alexa Fluor 568-conjugated goat anti-mouse IgG (1:1000; Thermo Fisher Scientific, Waltham, MA, UCA). Finally, the cells were stained with Hoechst 33342 (Chemical Dojin Co., Ltd., Kumamoto, Japan) to identify their nuclei. The vinculin distribution area was calculated using the ImageJ software.

### YAP Immunostaining

The cell treatment was performed identically to immunostaining vinculin to observe YAP accumulation. After blocking with 0.1% BSA to exclude the non-specific binding of antibodies, the cells were incubated in YAP polyclonal primary antibody (1:1000; Proteintech, USA) for 30 min. After incubation, the cells were washed with PBS and incubated with Alexa Fluor 488-conjugated goat anti-mouse IgG (1:2000; Thermo Fisher Scientific, Waltham, MA, UCA). Finally, the cells were stained with Hoechst 33342 (Chemical Dojin Co., Ltd., Kumamoto, Japan) to identify the nuclei. YAP was observed using a fluorescence microscope (Nikon Co., Ltd., Tokyo, Japan), and images were captured in a CCD camera and processed using the ImageJ software.

### Statistical analysis

Five samples (n = 5) were used for each analysis, and the data were presented as mean ± standard deviation. Additionally, the one-way analysis of variance with Tukey’s post-hoc test was used to examine the differences among the different groups. The p-values < 0.05 were considered statistically significant, with *, **, ***, and **** indicating p < 0.05, p < 0.01, p < 0.005, and p < 0.001, respectively. All statistical analyses were performed using the GraphPad Prism 9 software version 9.4.1.

## Results

### Characterization of disordered abrasive scratches on Ti

**Figure 1:**
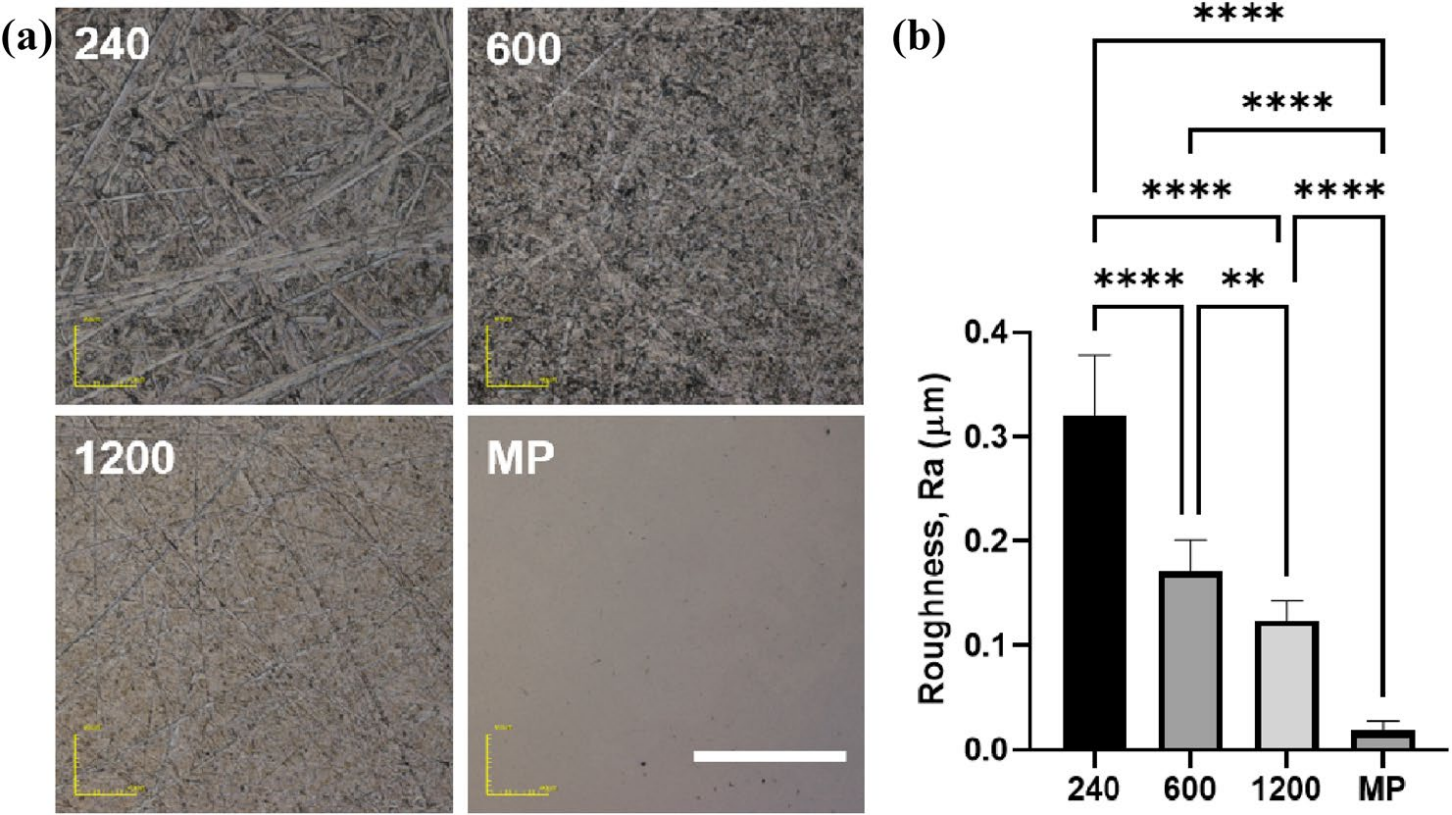
Confocal laser microscopic images (**a**) and surface roughness, *Ra* (**b**) of disordered abrasive scratches on Ti surface. The scale bar indicates 100 μm. The data shown are mean ± SD. **p < 0.01; ****p < 0.001.

In general, the polishing marks overlapped and formed a complex morphology on the Ti surface; the marks on specimen #240 were the largest. As the size of the abrasive grains in the polishing paper decreased, the size of the polishing marks reduced. Additionally, the marks were straight despite the disordered orientation (Fig. 1a). No polishing marks were observed on the MP surfaces. The surface roughness (*Ra*) values for specimens #240, #600, #1200, and MP were 0.32, 0.17, 0.12, and 0.02 μm, respectively. Figure 1b indicates that the *Ra* value depended on the size of the polishing marks on the surface.

### Cell shape and actin fiber formation on Ti with disordered abrasive scratches

**Figure 2:**
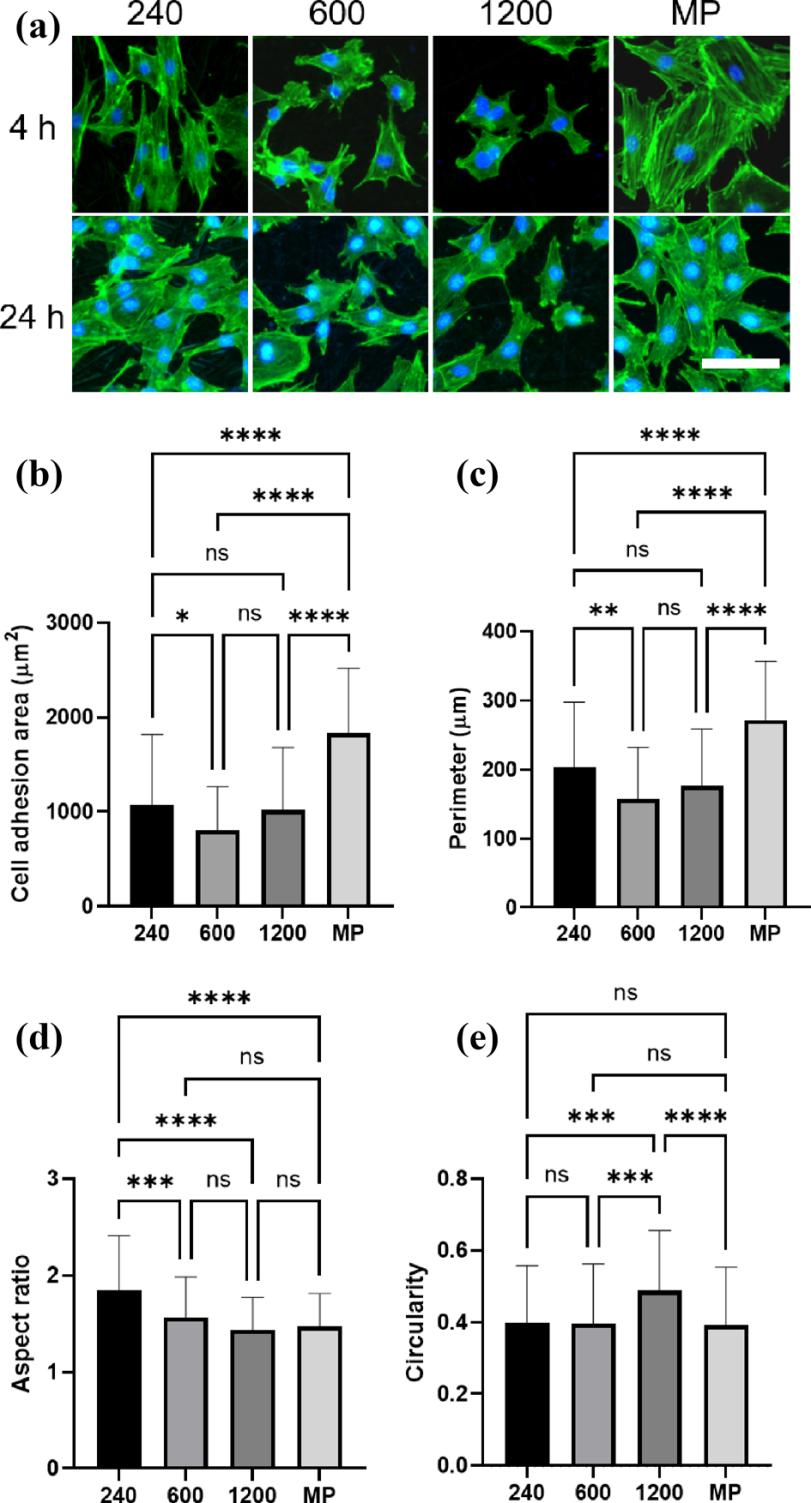
Cell attachment behavior on Ti with disordered abrasive scratches. Fluorescent microscopic images of the cells cultured on the Ti with disordered abrasive scratches for 4 h and 24 h (a). The cells-stained F-actin (green) and nuclei (blue). The scale bar indicates 100 μm. Cell morphological parameter of cell attachment area (**a**), perimeter (**b**), aspect ratio (**c**), and circularity (**d**) calculated from the images of 4 h cultivation. The data shown are mean ± SD. *p < 0.05; **p < 0.01; ***p < 0.005; and ****p < 0.001.

Figure 2A depicts the morphologies of the cells cultured on the specimens for 4 and 24 h. Clear actin stress fibers were observed in cells cultured on specimens #240 and MP for 4 h, whereas unclear bundles were observed in those cultured on specimens #600 and #1200. After 24 h of cultivation, prominent bundles were observed on the #240 and MP surfaces; however, smaller and fewer bundles were observed on the #600 and #1200 surfaces. Figure 2b shows the morphological analysis of the cells, including the cell adhesion area, perimeter, circularity, and aspect ratio. The cell adhesion areas of the #600 and #1200 surfaces were smaller than that of the #240. Additionally, the cells exhibited the largest spreading area on the MP surface. The presence of polishing marks decreased the cell spreading area. The circularities of the #600 and #1200 surfaces were higher than those of the #240 and MP surfaces. As circularity indicated the ratio of area and perimeter, the cells cultured on specimens #600 and #1200 exhibited poor pseudopodium and were round-shaped. These results indicated that osteoblasts exhibited a smaller adhesion area and tended to form a round shape on the #600 and #1200 surfaces. Conversely, the surface of specimen #240 induced a high aspect ratio, and the cells formed a spindle shape.

### Focal adhesion of cells cultured on Ti with disordered abrasive scratches

**Figure 3:**
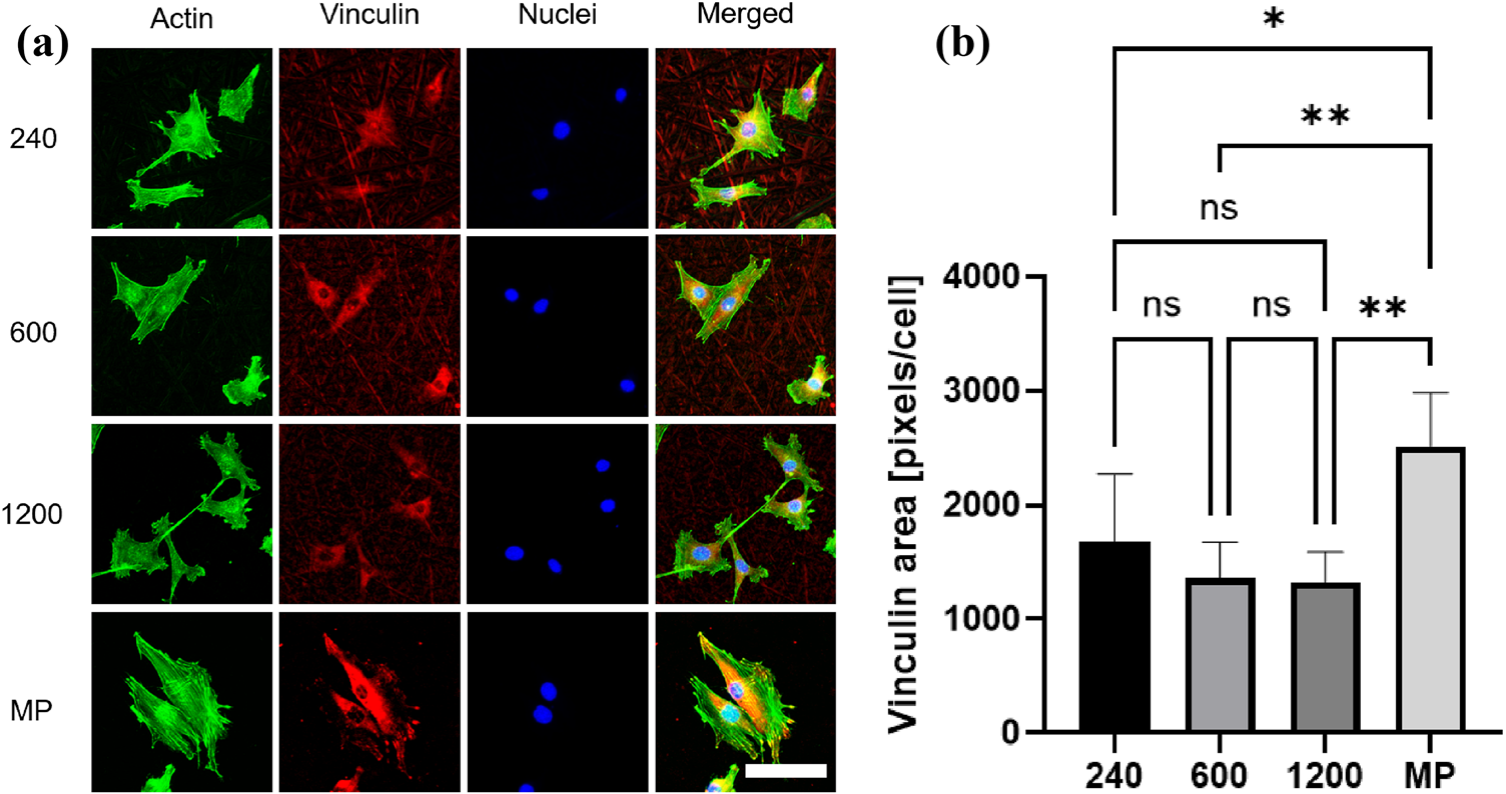
Vinculin expression of cells cultured on Ti with disordered abrasive scratches. Fluorescent microscopic images of F-actin and vinculin (**a**) and image base analysis of vinculin expression (**b**). The scale bar indicated 100 μm, and the data shown are mean ± SD. *p < 0.05; **p < 0.01. ns indicates no significant difference.

Figure 3A depicts the images of vinculin immunostaining of the actin cytoskeleton. The levels of vinculin on the cells cultured on the MP surface were remarkably higher than those observed on the cells cultured on the #240, #600, and #1200 surfaces. Moreover, vinculin accumulation at the tip of the stretched cytoplasm appeared only on the MP surface (Fig. 3a). Figure 3b shows the image-based analysis of vinculin expression per cell. The cells cultured on the MP surface exhibited greater vinculin expression than those cultured on the other surfaces. Furthermore, disordered abrasive scratches tended to induce lower vinculin expression in osteoblasts.

### YAP distribution of the cells on Ti with disordered abrasive scratches

**Figure 4:**
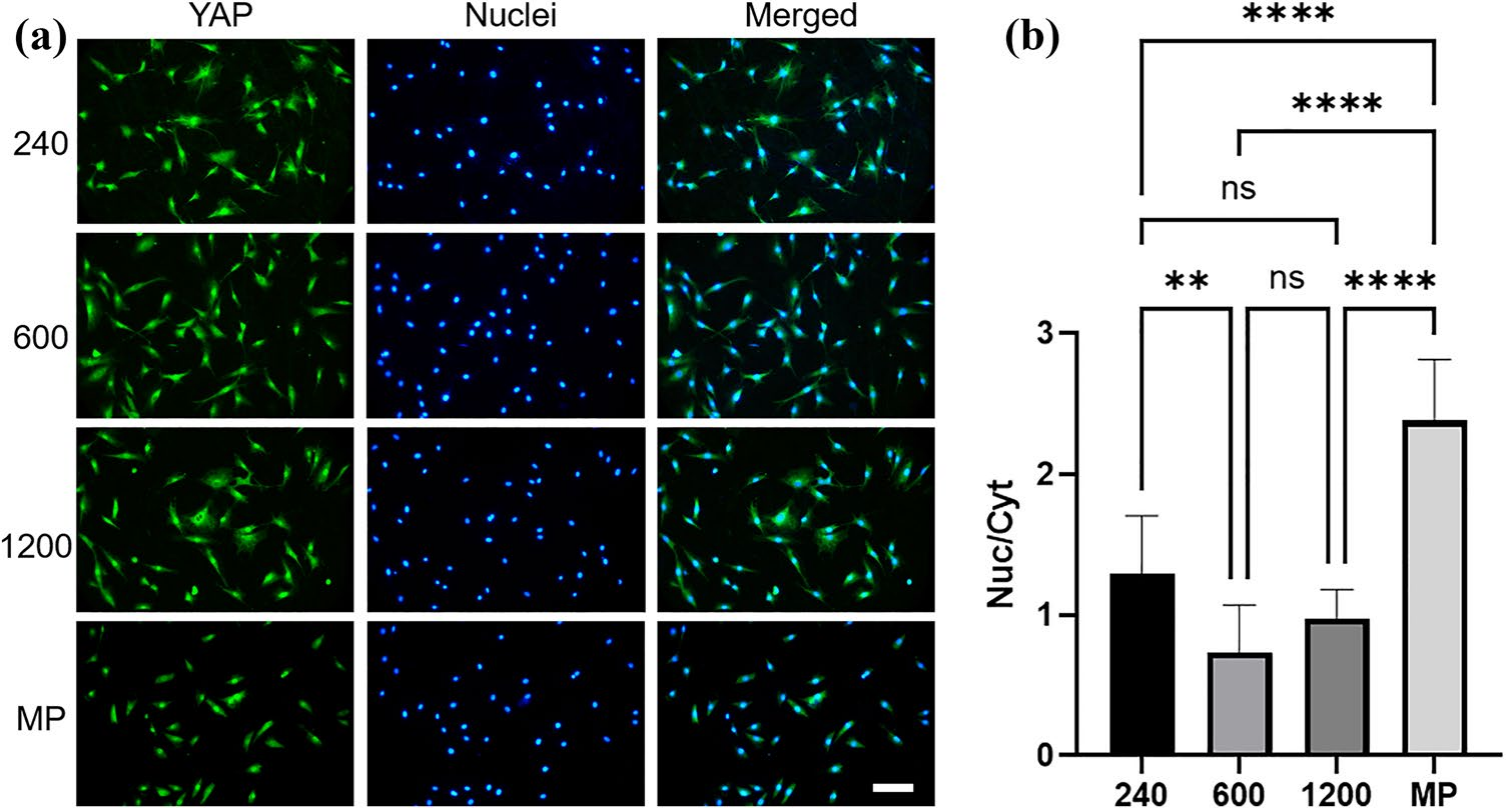
YAP distribution of cells cultured on Ti with disordered abrasive scratches. Fluorescent microscopic images of YAP (**a**) and its distribution of nucleus to cytoplasm ratio (**b**). The scale bar indicated 100 μm, and the data shown are mean ± SD. **p < 0.01; ****p < 0.001. ns indicates no significant difference.

Figure 4 illustrates the YAP distribution ratio in the nucleus and cytoplasm. The YAP distribution ratio (Nuc/Cyt) was approximately 1.0 for the cells cultured on the #600 and #1200 surfaces. Conversely, the value of Nuc/Cyt was approximately 2.0 for the cells cultured on the MP surface. The cell spreading area of the specimens determined the YAP distribution. The YAP was translocated in the nucleus for large cell areas, whereas the YAP was retained in the cytoplasm for small cell areas.

## Discussion

This study focused on revealing a regulation mechanism of cell proliferation and differentiation on disordered abrasive scratches. The surface roughness and surface morphology are expected to affect cellular function. The cell spreading inhibited on the disordered grooves in our previous study. Therefore, we assumed that the cells sensed surface morphology through cell spreading.

Integrins, a receptor at the plasma membrane, acts as the linker between cells and extracellular matrix. Hence, surface roughness affects the integrin expression profile. Olivares-Navarrete et al. claimed that the $\alpha$_2_β_1_ integrin plays a critical role in adhesion on the Ti surface with micron-scale and submicron-scale structure^23^. Typically, nano/micro-roughness of the Ti surface promotes the differentiation of osteoblasts. The optimized surface profile for triggering the integrin activation and cell differentiation is a nano/micro-hybrid roughened surface with a height of 2–4 nm, which induces rearrangement of the cytoskeleton^24^. However, it is unknown why the surface induces the specific integrin subclass.

Ordered microgrooves could induce rearrangement of cytoskeleton. Anisotropic morphology, such as microgrooves, induce contact guidance^25^, which involves cell alignment and migration along the direction of the anisotropy. On the microgrooves, alignment of microtubules, rearrangement of actin microfilament bundles, and alignment of focal contacts were observed after 3 h of cell seeding, in this order^26^. Huang et al. claimed that the cell attachment rate increased on ordered grooves with approximately *Ra* = 150 nm^27^. A Similar result has been reported^28^. In addition, Chen et al. claimed that ordered micro/nano grooved surfaces created by femtosecond laser technique promote osteogenic differentiation and calcification, although the cell attachment and proliferation on the surface were less than that on the mirror-polished titanium^29^.

In contrast, the effect of disordered grooves on cellular function is yet to be precisely explored. Previously, we reported that the Ti surface, which has disordered abrasive scratches with approximately Ra = 100 nm, inhibits cell attachment and osteogenic differentiation^19^. Linez-Bataillon et al. reported similar results, wherein mirror-polished Ti exhibited higher cell attachment than roughened Ti^30^. The disordered grooves, that is, isotropic morphology, induced disordered contact guidance along the grooves and random cell attachment without cell elongation^31, 32^. In this study, a submicron-roughened surface was produced by polishing, which resulted in disordered abrasive scratches on the surface (Fig. 1). These disordered abrasive scratches induced multiple contact guidance to the cells, which eliminated anisotropy from the cells. The cells exhibited contact guidance along a part of the polishing marks; however, cell spreading was inhibited because the marks were disordered and overlapped on the surface (Figs. 2 and 3). Therefore, clear actin filaments were not observed on the disordered abrasive scratches.

Cell spreading area relates to YAP distribution. The transcription factor YAP/TAZ can sense the extracellular microenvironment and serve as a mediator of mechanotransduction^33^. Wada et al. reported that the stress fibers increased with the expansion of the cell spreading area, and the YAP downstream of cell morphology was regulated by F-actin^34^. The spreading cell area on the specimens determined the YAP distribution (Fig. 4); the YAP was translocated in the nucleus for large cell areas, whereas it was retained in the cytoplasm for smaller cell areas. Disordered abrasive scratches could inhibit cell spreading and actin fiber formation. Hence, YAP is retained in the cytoplasm. Then, cell proliferation and differentiation might be reduced by the disordered abrasive scratches. Results from our data indicate that the cells sense surface morphology by their spreading area.

Surface roughness and morphology is an important parameter for the successful implantation of medical devices. Xia et al. reported that pore size plays a more significant role than roughness in nanoporous polycarbonate membranes^35^. Although similar surface roughness was measured by atomic force microscopy, stem cell differentiation induced on larger pore surfaces was more significant than on smaller pore surfaces^19^. The surface morphology links to the adjusting procedure of the surface roughness, which varies in different studies. Our study findings indicated that surface morphology was more essential than roughness, at least the cellular level. Furthermore, disordered abrasive scratches on the Ti surface affected the cell attachment, regulation of F-actin formation, and the Hippo signaling pathway. However, the effect of the polishing marks in terms of width and depth remained unclear. In future, strictly regulated marks should be created on the Ti surface using nano-engineering methods, such as the femto-second laser technique, to verify the hypothesis involved in the process.

## Conclusion

To further understand the cell adhesion behavior, we investigated the cell spreading and YAP distribution on Ti surfaces with disordered abrasive scratches. The results indicated that larger polishing marks induced contact guidance, whereas smaller polishing marks eliminated the anisotropy from the cells and inhibited cell spreading. Furthermore, the YAP distribution was dependent on the cell-spreading area. Therefore, disordered abrasive scratches can regulate cell growth through the Hippo signaling pathway. In the future, we intend to examine the regulation of polishing marks to obtain a clearer understanding of the cell spreading hypothesis and its effect on nuclear translocation.

## Data Availability

The data that support the findings of this study are available from the corresponding author upon reasonable request.
